# Mid-trimester amniotic fluid proteome’s association with spontaneous preterm delivery and gestational duration

**DOI:** 10.1371/journal.pone.0232553

**Published:** 2020-05-07

**Authors:** Maria Hallingström, Petra Zedníková, Vojtěch Tambor, Malin Barman, Marie Vajrychová, Juraj Lenčo, Felicia Viklund, Linda Tancred, Hardis Rabe, Daniel Jonsson, Alisa Kachikis, Staffan Nilsson, Marian Kacerovský, Kristina M. Adams Waldorf, Bo Jacobsson

**Affiliations:** 1 Department of Obstetrics and Gynecology, Institute of Clinical Sciences, Sahlgrenska Academy, University of Gothenburg, Gothenburg, Sweden; 2 Department of Obstetrics and Gynecology, Sahlgrenska University Hospital, Gothenburg, Sweden; 3 Biomedical Research Center, University Hospital Hradec Kralove, Hradec Kralove, Czech Republic; 4 Department of Biological and Biochemical Science, Faculty of Chemical Technology, University of Pardubice, Pardubice, Czech Republic; 5 Department of Biology and Biological Engineering, Food and Nutrition Science, Chalmers University of Technology, Gothenburg, Sweden; 6 Department of Molecular Pathology and Biology, Faculty of Military Health Sciences, University of Defense, Hradec Kralove, Czech Republic; 7 Department of Analytical Chemistry, Faculty of Pharmacy, Charles University, Hradec Kralove, Czech Republic; 8 Stockholm South General Hospital, Stockholm, Sweden; 9 Biobank Väst, Sahlgrenska University Hospital, Gothenburg, Sweden; 10 Department of Infectious Diseases, Institute of Biomedicine, Sahlgrenska Academy, University of Gothenburg, Gothenburg, Sweden; 11 Department of Obstetrics and Gynecology, University of Washington, Seattle, Washington, USA; 12 Department of Mathematical Sciences, Chalmers University of Technology, Gothenburg, Sweden; 13 Department of Pathology and Genetics, Institute of Biomedicine, Sahlgrenska Academy, University of Gothenburg, Gothenburg, Sweden; 14 Department of Obstetrics and Gynecology, Charles University in Prague, Faculty of Medicine in Hradec Kralove, Hradec Kralove, Czech Republic; 15 Department of Genetics and Bioinformatics, Area of Health Data and Digitalisation, Institute of Public Health, Oslo, Norway; Shanghai Jiao Tong University, CHINA

## Abstract

**Background:**

Amniotic fluid is clinically accessible via amniocentesis and its protein composition may correspond to birth timing. Early changes in the amniotic fluid proteome could therefore be associated with the subsequent development of spontaneous preterm delivery.

**Objective:**

The main objective of this study was to perform unbiased proteomics analysis of the association between mid-trimester amniotic fluid proteome and spontaneous preterm delivery and gestational duration, respectively. A secondary objective was to validate and replicate the findings by enzyme-linked immunosorbent assay using a second independent cohort.

**Methods:**

Women undergoing a mid-trimester genetic amniocentesis at Sahlgrenska University Hospital/Östra between September 2008 and September 2011 were enrolled in this study, designed in three analytical stages; 1) an unbiased proteomic discovery phase using LC-MS analysis of 22 women with subsequent spontaneous preterm delivery (cases) and 37 women who delivered at term (controls), 2) a validation phase of proteins of interest identified in stage 1, and 3) a replication phase of the proteins that passed validation using a second independent cohort consisting of 20 cases and 40 matched controls.

**Results:**

Nine proteins were nominally significantly associated with both spontaneous preterm delivery and gestational duration, after adjustment for gestational age at sampling, but none of the proteins were significant after correction for multiple testing. Several of these proteins have previously been described as being associated with spontaneous PTD etiology and six of them were thus validated using enzyme linked immunosorbent assay. Two of the proteins passed validation; Neutrophil gelatinase-associated lipocalin and plasminogen activator inhibitor 1, but the results could not be replicated in a second cohort.

**Conclusions:**

Neutrophil gelatinase-associated lipocalin and Plasminogen activator inhibitor 1 are potential biomarkers of spontaneous preterm delivery and gestational duration but the findings could not be replicated. The negative findings are supported by the fact that none of the nine proteins from the exploratory phase were significant after correction for multiple testing.

## Introduction

One of the major problems in obstetrics is preterm delivery (PTD, < 37 weeks of gestation), with a global annual estimated incidence of 15 million and 1 million associated neonatal deaths [1]. PTD can either be iatrogenic (medically indicated) [2] or spontaneous in origin [3], where the latter accounts for 75% of all PTD [4]. Multiple distinct pathways and complex biological events within several compartments (maternal blood, genital tract, placenta, amniotic fluid and the fetus) contribute to the development of spontaneous PTD [5–9], but these are not yet fully elucidated.

Mid-trimester amniocentesis is performed for genetic testing and provides a unique window into the amniotic fluid milieu of women with normal pregnancies and pregnancies destined to deliver preterm. The amniotic fluid surrounds the developing fetus [10] and is in close proximity to the placenta, which reflects a steadily developing organ with a major impact on birth timing [11]. Proteomic profiling of the amniotic fluid composition in early gestation may provide insight into basic biological mechanisms, detect different pathological conditions [12] and increase our understanding of biological factors that contribute to gestational duration. Furthermore, it may identify early biomarkers of spontaneous PTD and increase our knowledge about associated pathways [13–16].

Development of a suite of “-omics” platforms, such as genomics, proteomics and metabolomics, have enabled the unbiased discovery of complex networks associated with healthy and abnormal pregnancies [14, 17]. Proteomics provides a unique opportunity to explore the whole proteome encoded by the genome [18, 19]. A large number of differences in the proteins quality, quantity [20], function and structure can be characterized [18, 19], which provides a more comprehensive picture of biological events [21] during gestation. Proteomics has previously been used to diagnose intra-amniotic inflammation in women with subsequent spontaneous PTD [22–24] as well as in women with symptoms of preterm labor (PTL) and preterm prelabor rupture of membranes (PPROM) at risk for spontaneous PTD [25]. Furthermore, proteomics has enabled the identification of infectious pathways associated with histologic chorioamnionitis in women with PPROM [26, 27]. However, only a few studies have analyzed the mid-trimester amniotic fluid proteome in relation to spontaneous PTD in singletons [28, 29]. Fotopoulou et al. identified seven clusters on three types of protein chips that were significantly different between women with a subsequent spontaneous PTD and women who delivered at term. However, they were unable to identify the individual proteins or peptides of these clusters [28]. In a prior study, our group used a pooled sample strategy in which C-reactive protein emerged as a significant and interesting candidate, but results in the validation phase were non-significant [29].

The ability to predict women at increased risk for spontaneous PTD remains poor despite extensive research efforts [14]. The main objective of this study was to perform unbiased proteomics analysis of the mid-trimester amniotic fluid proteome and to investigate its association with subsequent spontaneous PTD and gestational duration. A secondary aim was to validate and replicate the findings using a second independent cohort, analyzed by enzyme-linked immunosorbent assay (ELISA).

## Materials and methods

### Description of the cohort

A total of 1546 women underwent transabdominal amniocentesis for genetic purposes at 14–19 weeks of gestation at Sahlgrenska University Hospital/Östra, Gothenburg, Sweden between September 2008 and September 2011. Clinical indications for amniocentesis were advanced maternal age, abnormal first-trimester combined screening, family history of chromosomal abnormalities or genetic diseases, or anxiety about potential chromosomal abnormalities. Women were offered enrollment if they were ≥ 18 years of age and had a viable singleton pregnancy. Women were ineligible (*n* = 425) for the following reasons: i) infection with human immunodeficiency virus, hepatitis B virus or hepatitis C virus, ii) suspected or confirmed congenital fetal abnormality, and/or iii) clinical amniocentesis at times prior to study start. Women were excluded from the study (*n* = 497) if they, i) declined participation, ii) were unable to understand the written and oral information in Swedish, iii) had an insufficient amniotic fluid sample, or iv) had an initial twin gestation with fetal demise of one twin or with vanishing twin.

Medical records were abstracted from all eligible subjects (*n* = 624) at inclusion and after delivery to obtain information on subjects’ demographics and maternal health, as well as pregnancy, delivery and neonatal outcomes. Women who had a termination of pregnancy (*n* = 12) or were lost to follow-up (*n* = 6) were excluded. Women were also excluded with certain maternal diseases (e.g. hypo- and hyperthyroidism, diabetes mellitus, preeclampsia, uterine anomaly, multiple sclerosis, autoimmune system diseases, rheumatism, ulcerative colitis, hemostatic disorders, syphilis, renal disease, leukemia, severe asthma, hypertension, hereditary chromosomal defects, vitamin D deficiency and epilepsy), fetal complications (congenital anomaly and fetal growth restriction), and sampling or analytical deviations (discolored amniotic fluid at sampling due to blood contamination) and where sample handling deviated from the study protocol.

### Cohort selection for the exploratory and validation phases

**Fig 1:**
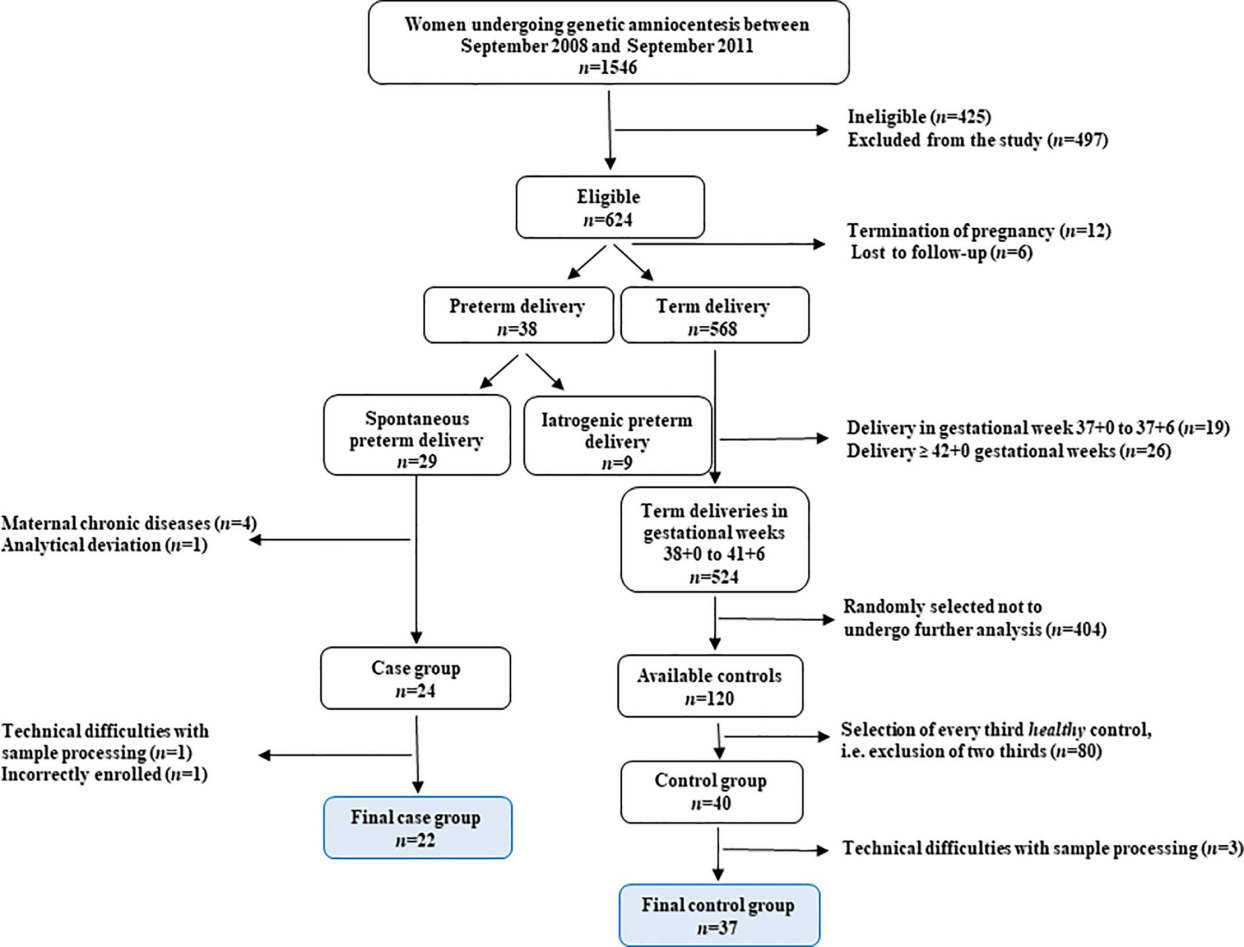
Selection and application of inclusion and exclusion criteria to subjects in the cohort. This flowchart illustrates the selection and application of inclusion and exclusion criteria to subjects of the cohort.

Women were categorized into two groups; women with PTD (*n* = 38) and women with a term delivery (*n* = 568). From the group of women with a PTD, women with iatrogenic PTD (*n* = 9), and women who fulfilled the exclusion criteria described above (*n* = 6) were excluded, leaving 23 women with a spontaneous PTD to constitute the case group. To achieve a greater separation in phenotype between cases and controls and a more homogenous control group, the group of women with a delivery at term were limited to women delivering between 38+0 and 41+6 gestational weeks (*n* = 524). A random selection of 120 controls was then performed and exclusion criteria applied; from this group, every third subject was selected to constitute the final control group. During the proteomic analytical stage, one case of spontaneous PTD and 3 controls had to be excluded due to a broken injection needle in the HPLC system and other technical difficulties with sample processing, leaving 22 cases and 37 controls in the final study cohort that would be used for the exploratory and validation phases (Fig 1).

### Cohort selection for the replication phase

For the replication phase, a second independent cohort of amniotic fluid samples was used from women undergoing genetic amniocentesis at 14–19 weeks gestation at Sahlgrenska University Hospital/Östra, Gothenburg, Sweden between September 2008 and July 2017. This cohort included 24 women with spontaneous PTD; 23 women who were identified in the second cohort and the single spontaneous PTD case from the exploratory phase cohort that was not analyzed by proteomics, due to technical difficulties with sample processing. Eligibility and exclusion criteria as described above were applied and four cases of spontaneous PTD were excluded, leaving 20 cases of spontaneous PTD in the final case group of the replication phase. Cases were matched to healthy controls in a 1:2 ratio using the following criteria; a) maternal age at sampling (± 2 years), b) parity (primiparous/multiparous), c) gestational age at sampling (± 3 days), and d) whether the pregnancy was conceived using in vitro fertilization (IVF; yes/no), choosing the preceding and subsequent control based on date of sampling. Matching was successful except for three cases where gestational age at sampling had to be extended by one to two days for the matched controls. There was no overlap in regards to the study cohorts.

### Sample collection

Ultrasound-guided transabdominal amniocentesis was performed on all participants and an additional three milliliters (mL) of amniotic fluid was collected for the purposes of this study. The amniotic fluid was immediately stored at 4–8°C until processing. Samples were then centrifuged for 20 minutes at 12,000 *g* at 4°C to remove cells and debris. Pellets and supernatants were frozen at -80°C awaiting analysis.

### Ethics statement

The study was approved by the Central Ethics Review Board at the University of Gothenburg, Sweden (Dnr Ö 639–03, T 318–08, T 694–11). Written informed consent was obtained from all women who met inclusion criteria and agreed to participate.

### Amniotic fluid assessment

**Fig 2:**
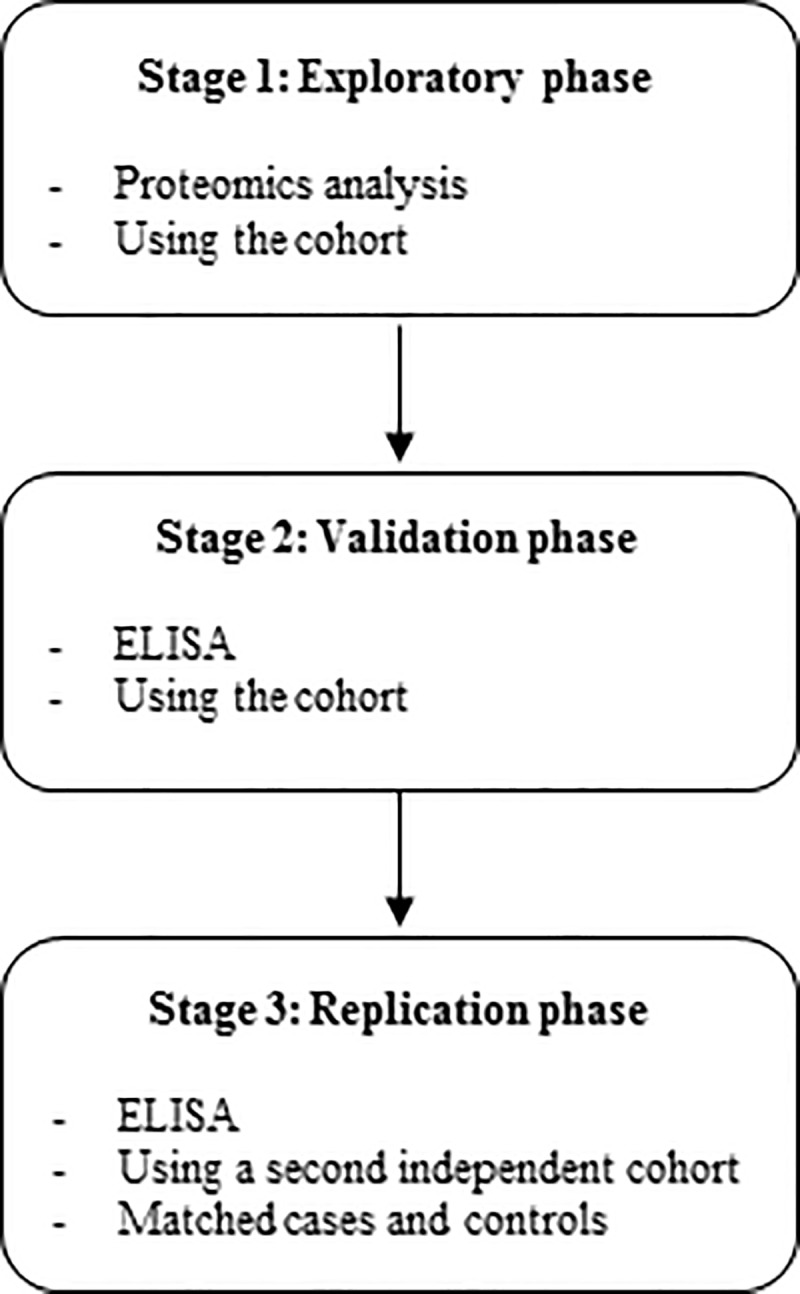
Schematic overview of the three analytical stages. Samples were initially analyzed in the exploratory phase by proteomics. Significant proteins were validated by ELISA using the same cohort of samples. Successfully validated proteins alterations were subjected for replication using a second independent cohort.

Fig 2 outlines the study design beginning with proteomics analyses (stage 1, exploratory phase), followed by ELISA validation (stage 2, validation phase) and then determining whether the proteins successfully validated were differentially expressed in a second, independent cohort (stage 3, replication phase).

#### Stage 1: Exploratory phase—proteomic analysis

In brief, individual samples were immunodepleted and digested with trypsin and lys C. Individual amniotic fluid samples were tagged and multiplexed using iTRAQ reagents, which are amine-specific and can label four biological samples to enable simultaneous identification and quantitation within pooled multiplexed samples. The digested and tagged peptide samples were subjected to high pH fractionation and each fraction was analyzed using reverse phase high performance liquid chromatography (LC)-mass spectrometry (MS) hyphenated to MS (S1 File and S1 Fig).

Each sample was supplemented with protease inhibitors (Complete Mini, EDTA-free Protease Inhibitor Cocktail; Roche Diagnostics, Basel, Switzerland) and filtered using centrifugal filterswith 0.22 μm PVDF membrane (Merck Millipore, Bedford, USA) to remove potential particulate matter. Total protein concentration was determined using the BCA Protein Assay Kit (Sigma Aldrich, USA). An equal amount of protein was taken from each sample for immunodepletion of 14 abundant plasma proteins using 4.6 × 100 mm column Multiple Affinity Removal System (MARS) Hu-14 (Agilent, Palo Alto, USA) according to the manufacturer’s instructions. Samples were supplemented with 0.1% Rapigest (Waters Milford, USA), buffered with triethylammonium bicarbonate, pH 8,5 (Sigma Aldrich, St. Louis, USA), reduced by 5 mM tris-(2-carboxyethyl)phosphine (Sigma Aldrich, St. Louis, USA) and the thiol groups were blocked with S-methyl methanethiosulfonate (Sigma Aldrich, St. Louis, USA). Protein samples were digested with LysC (Promega, Madison, USA) for 4 hours at 37°C. Subsequently, trypsin (Promega, Madison, USA) was added and proteins were digested overnight.

An iTRAQ 4-plex kit (AB Sciex, Foster City, USA) was used to prepare 20 multiplexed samples. Each multiplex contained a global internal standard, pool of all samples, at the 114 iTRAQ channel and three individual amniotic fluid samples at iTRAQ channels 115, 116 and 117. The high pH fractionation of multiplexed samples was performed on an UltiMate3000 HPLC analytical system (Thermo Scientific, Bremen, Germany). Peptides were injected in mobile phase A (2% ACN, 20 mM ammonium formate) on a 2.1 × 100 mm column Xterra MS C18, 3.5 μm (Waters, Milford, USA) and separated at 0.3 mL/min using a 40-min gradient from 3 to 50% mobile phase B (80% ACN, 20 mM ammonium formate) into 32 subfractions collected between 6–30 minutes. The subfractions were pooled into 8 fractions and analyzed using an UltiMate 3000 RSLCnano system connected to a Q-Exactive Plus mass spectrometer (Thermo Scientific, Bremen, Germany). Peptides were loaded in 2% ACN, 0.1% TFA onto a PepMap100 ViperTrap 3 μm (Thermo Scientific, Bremen, Germany) pre-column and eluted on a 0.075 × 500 mm analytical column Acclaim Pep Map RSLC C18, 2 um (Thermo Scientific, Bremen, Germany) using a gradient formed by mobile phase A (2% ACN, 0.1% FA) and mobile phase B (80% ACN, 0.1% FA). Gradient run from 6 to 44% of mobile phase B in 60 minutes at the flow rate of 200 nl/min. The MS analysis was performed in the information dependent acquisition mode using the ten most intensive precursors for fragmentation (MS2). All samples were analyzed in technical triplicates. Peptide identification and quantitation were conducted in the Max Quant software version 1.5.2.8 using the reverse decoy mode and integrated false discovery rate analysis. The data was searched against the UniProtKB/Swiss-Prot database (human, canonical included, download September 3, 2015). Intensities of iTRAQ reporter ions were corrected using isotope factors supplied with the iTRAQ kit. The sample ratio to global internal standard was calculated from all reporter ion intensities. Proteins identified by the reverse database and potential contaminants were further filtered in order to obtain the final dataset.

#### Stages 2 and 3: The validation and replication phases

ELISA kits were chosen with a working range to include the most likely concentration ranges in amniotic fluid for each protein. Second, ELISA kits were tested to determine if matrix interference might occur from another protein present in amniotic fluid that could confound results interpretation. In three cases (insulin-like growth factor-binding protein 5 (IGFBP-5), insulin-like growth factor-binding protein 7 (IGFBP-7) and semaphorin-3B (SEMA A (V))), either linear dilution and/or spike-and-recovery approaches yielded non-linear results, indicating that there was specific interference by a factor present within the amniotic fluid samples (Human IGFBP-5, (KA1727) Novus Biologicals, Littleton, CO, USA; Human IGFBP-7 (NBP2-62762) Novus Biologicals Littleton, CO, USA; Human SEMA3B/SemA (EKH4388) Nordic Biosite, Täby, Sweden). Therefore, efforts were concentrated on validating candidate proteins where it could be confirmed that the commercially available ELISA kits yielded quantitative and replicable results: extracellular superoxide dismutase [Cu-Zn] (EC-SOD), lipocalin-15 (LCN15), microfibril-associated glycoprotein 4 (MFAP4), neutrophil gelatinase-associated lipocalin (NGAL), plasminogen activator inhibitor 1 (PAI-1) and urotensin-2 (U-II), with the proteins short protein/gene name within parenthesis (S1 Table).

Amniotic fluid samples were analyzed in duplicate within 24 hours of thawing. Analyses were performed according to the manufacturer’s instructions and laboratory personnel were blinded to case-control status and other clinical data. Amniotic fluid dilutions used and the working range of each kit (S2 Table) were as follows: EC-SOD (dilution 1:50; standard curve range 0.625–40 ng/mL), LCN15 (dilution 1:10; standard curve range 18.75–1200 pg/mL), MFAP4 (dilution 1:10; standard curve range 0.313–20 ng/mL), NGAL (dilution 1:200; standard curve range 0.003–0.040 ng/mL), PAI-1 (dilution 1:10; standard curve range 0.31–20 ng/mL) and U-II (dilution 1:20; standard curve range 15.6–1000 pg/mL). Samples found to be above or below the detection limit were re-analyzed in a higher or lower sample dilution. Candidate proteins alterations that were successfully validated, were subjected to replication.

### Statistical analyses

Subject characteristics were compared using Mann Whitney *U* test, Chi-Square and Fisher’s exact test. Pearson correlation was used to analyze the correlation between log transformed protein concentration or intensity ratio and gestational age at sampling and gestational duration, respectively, for both cohorts. Student’s t-test or analysis of covariance (ANCOVA) were used to compare means of log transformed protein concentrations between cases and controls in the cohort used for the exploratory and validation phases. A linear mixed model was used in the second cohort of matched data for the replication phase. The significance level was set to *p*<0.05 using a two-sided alternative hypothesis. All statistical analyses were performed in SPSS 25.0 for Windows (SPSS Inc, USA) and R version 3.3.1. The data was graphically displayed using the Software Perseus version 1.5.1.6.

## Results

### Stage 1: The exploratory phase

Maternal and neonatal characteristics of the cohort used in the exploratory and validation phases (stages 1 and 2) are presented in Table 1. Women delivering preterm were significantly older than the women delivering at term, but otherwise there were no significant differences between the groups for parity, use of in vitro fertilization (IVF), body mass index (BMI), smoking, rate of previous PTD, gestational age at sampling, mode of delivery, neonatal sex or Apgar score.

**Table 1:** Maternal and neonatal characteristics of the cohort.

Variable	Spontaneous preterm delivery (*n* = 22)	Term delivery (*n* = 37)	*p*
Gestational duration (weeks+days)	35+5 (33+5–36+5)	40+1 (39+2–41+1)	
Birth weight (grams)	2575 (2358–2870)	3540 (3260–3778)	
Maternal age at sampling (years)	38 (36–41)	36 (35–38)	**0.029**
Nulliparous	14 (63.6%)	27 (73.0%)	0.451
IVF	4 (18.2%)	1 (2.7%)	0.059
Maternal BMI at first prenatal visit	24.6 (23.6–26.9)	23.6 (20.9–26.6)	0.215
Smoking at first prenatal visit	2 (9.1%)	1 (2.7%)	0.549
Previous preterm delivery	3 (13.6%)	1 (2.7%)	0.141
Gestational age at sampling (weeks+days)	15+4 (15+0–16+2)	15+5 (15+2–16+2)	0.460
Mode of delivery			
Vaginal delivery	19 (86.4%)	28 (75.7%)	0.506
Vacuum extraction	0 (0.0%)	3 (8.1%)	0.286
Cesarean section	3 (13.6%)	6 (16.2%)	1.000
Neonatal sex			0.564
Male	9 (40.9%)	18 (48.6%)	
Female	13 (59.1%)	19 (51.4%)	
Apgar score < 7 at 5 min	0 (0.0%)	2 (5.4%)	0.524

Continuous variables were analyzed using a Mann-Whitney *U* Test and are presented as the median (interquartile range; IQR). Categorical variables were analyzed using Pearson Chi-Square or Fisher’s Exact Test (when ≤5 individuals) and are shown as N (%).Bold text indicate statistical significance at p<0.05 using a two-sided alternative hypothesis.

A total of 43,854 MS/MS spectra were identified with an average number of 3,185 proteins per multiplex after filtering for reverse and contaminant proteins. Next, selection criteria were applied to identify the most likely protein candidates in mid-trimester amniotic fluid that may be correlated with spontaneous PTD or gestational duration. The final data set after filtering consisted of 1,111 quantified proteins with at least 2 valid values out of 3 and CV < 20% in at least 75% in both groups. After filtering, 8% of the data were missing and were imputed before normalization using the mean of data in replicates (if present) or with median of the total data set. After imputation, data were subjected to LOESS normalization in R. In order to maximize the chances of identifying proteins correlated to spontaneous PTD, data was filtered to require a difference of intensity ratio between cases and controls of |Δ|≥0.2. A total of 33 proteins met these filtering criteria (S3 Table).

Analysis of crude data, adjusting for gestational age at sampling, revealed significantly higher concentrations of LCN15 (Δ = 0.41, *p* = 0.039) and MFAP4 (Δ = 0.23, *p* = 0.040) and significantly lower concentrations of EC-SOD (Δ = -0.26, *p* = 0.004), IGFBP-5 (Δ = -0.22, *p* = 0.046), IGFBP-7 (Δ = -0.32, *p* = 0.043), NGAL (Δ = -0.28, *p* = 0.044), PAI-1 (Δ = -0.29, *p* = 0.001), SEMA A (V) (Δ = -0.23, *p* = 0.001) and U-II (Δ = -0.22, *p* = 0.009) in women with spontaneous PTD compared to women who delivered at term. Furthermore, a significant negative correlation was observed between LCN15 (r = -0.345, *p* = 0.009) and MFAP4 (r = -0.334, *p* = 0.011) concentrations and gestational duration, while a significant positive correlation was seen for EC-SOD (r = 0.385, *p* = 0.003), IGFBP-5 (r = 0.291, *p* = 0.029), IGFBP-7 (r = 0.317, *p* = 0.017), NGAL (r = 0.289, *p* = 0.030), PAI-1 (r = 0.426, *p* = 0.001), SEMA A (V) (r = 0.375, *p* = 0.011), and U-II (r = 0.312, *p* = 0.019) concentrations, respectively (Table 2). Notably, maternal age was not significantly associated with any of the final protein concentrations. None of the proteins were significant after correction for multiple testing.

**Table 2:** The final proteins from the proteomic analysis.

Protein	Short protein/gene name	Primary accession number	Δ	*p*_Δ_	r	*p*_*r*_
*Increased Protein Expression*						
Lipocalin-15	LCN15	Q6UWW0	0.41	0.039	-0.345	0.009
Microfibril-associated glycoprotein 4	MFAP4	P55083	0.23	0.040	-0.334	0.011
*Decreased Protein Expression*						
Extracellular superoxide dismutase [Cu-Zn]	EC-SOD	P08294	-0.26	0.004	0.385	0.003
Insulin-like growth factor-binding protein 5	IGFBP-5	P24593	-0.22	0.046	0.291	0.029
Insulin-like growth factor-binding protein 7	IGFBP-7	Q16270	-0.32	0.043	0.317	0.017
Neutrophil gelatinase-associated lipocalin	NGAL	P80188	-0.28	0.044	0.289	0.030
Plasminogen activator inhibitor 1	PAI-1	P05121	-0.29	0.001	0.426	0.001
Semaphorin-3B	SEMA A (V)	Q13214	-0.23	0.001	0.375	0.011
Urotensin-2	U-II	O95399	-0.22	0.009	0.312	0.019

The table presents the nine differentially expressed proteins by proteomics analysis that were nominally significantly correlated with both spontaneous PTD and gestational duration, adjusted for gestational age at sampling.

Δ reflects the difference of intensity ratio between cases and controls and *p*_**Δ**_ is the corresponding *p* value. r is the correlation between intensity ratio and gestational duration adjusted for gestational age at sampling and *p*_***r***_ is the corresponding *p* value.

### Stage 2: The validation phase

Next, ELISA was used to validate the findings from the proteomics analysis. Significantly decreased concentrations of MFAP4 (spontaneous PTD: median 6.9 ng/mL (IQR 4.2–8.1) vs term: median 8.5 ng/mL (IQR 5.6–19.2); *p* = 0.005), NGAL (spontaneous PTD: median 224.0 ng/mL (IQR 129.3–360.0) vs term: median 372.0 ng/mL (IQR (212.1–527.5); *p* = 0.011) and PAI-1 (spontaneous PTD: median 26.8 ng/mL (IQR (20.6–39.5) vs term: median 38.2 ng/mL (IQR 26.5–80.0); *p* = 0.010) in women with a spontaneous PTD were observed in the validation phase. NGAL concentrations were also associated with gestational age at sampling (*p* = 0.038) and adjusted accordingly (S4 Table). Validation of MFAP4 showed an opposite effect compared to the exploratory phase with significantly lower concentrations in women with spontaneous PTD compared to women with a term delivery during validation. No differences were observed between cases and controls in the validation phase for levels of EC-SOD (spontaneous PTD: median 51.5 ng/mL (IQR 41.3–60.0) vs term: median 50.5 ng/mL (IQR 38.8–61.3); *p* = 0.367), LCN15 concentrations (spontaneous PTD: median 347.5 pg/mL (IQR 283.1–419.0) vs term: median 318.8 pg/mL (IQR 262.6–377.5); *p* = 0.259), or U-II (spontaneous PTD: median 40.5 pg/mL (IQR 28.0–66.4) vs term: median 39.3 pg/mL (IQR 25.8–90.6); *p* = 0.317) (Table 3).

**Table 3:** Distribution of protein concentrations among cases and controls in the validation phase.

Short protein/ gene name	Spontaneous preterm delivery (*n* = 22)	Term delivery (*n* = 37)	*p*
EC-SOD (ng/mL)	51.5 (41.3–60.0)	50.5 (38.8–61.3)	0.367
LCN15 (pg/mL)	347.5 (283.1–419.0)	318.8 (262.6–377.5)	0.259
MFAP4 (ng/mL)	6.9 (4.2–8.1)	8.5 (5.6–19.2)	**0.005**
NGAL (ng/mL)	224.0 (129.3–360.0)	372.0 (212.1–527.5)	**0.011**
PAI-1 (ng/mL)	26.8 (20.6–39.5)	38.2 (26.5–80.0)	**0.010**
U-II (pg/mL)	40.5 (28.0–66.4)	39.3 (25.8–90.6)	0.317

Protein concentrations are shown as median (interquartile range; IQR). *p* values are obtained with Student’s t-test (or for NGAL with ANCOVA adjusting for gestational age at sampling) on log transformed values. Bold text indicates statistical significance at *p*<0.05 using a two-sided alternative hypothesis and only the significantly altered proteins have been replicated.

In the exploratory phase, a positive correlation was observed between EC-SOD, NGAL, PAI-1 and U-II concentrations and gestational duration. This correlation was confirmed in the validation phase for NGAL (r = 0.28, *p* = 0.033) and PAI-1 (r = 0.28, *p* = 0.031), but not for EC-SOD (r = -0.11, *p* = 0.390) and U-II (r = 0.18, *p* = 0.172) (Table 4). Finally, the negative correlation of LCN15 and MFAP4 concentrations and gestational duration observed in the exploratory phase was not verified in the validation phase; LCN15 (r = -0.12, *p* = 0.364) and MFAP4 (r = 0.33, *p* = 0.011), where MFAP4 again showed an opposite effect compared to the exploratory phase.

**Table 4:** Correlation between protein concentrations and gestational duration in the validation phase.

Short protein/ gene name	r	*p*
EC-SOD	-0.11	0.390
LCN15	-0.12	0.364
MFAP4	0.33	**0.011**
NGAL	0.28	**0.033**
PAI-1	0.28	**0.031**
U-II	0.18	0.172

Pearson correlation (r) between gestational duration and log transformed protein concentrations controlling for gestational age at sampling for NGAL. Bold text indicates statistical significance at p<0.05 using a two-sided alternative hypothesis.

### Stage 3: The replication phase

Maternal and neonatal characteristics of the second independent cohort used for the replication phase (Stage 3) are presented in Table 5. In this second cohort, there were no significant differences between women with a spontaneous PTD and their matched controls delivering at term, except the expected difference in gestational duration and birth weight. There were also no significant differences in characteristics between the cohort used in the exploratory and validation phases (Stages 1 and 2) and the second cohort used in the replication phase (Stage 3) (S5 Table).

**Table 5:** Maternal and neonatal characteristics of the second cohort.

Variable	Spontaneous preterm delivery (*n* = 20)	Term delivery (*n* = 40)	*p*
Gestational duration (weeks+days)	36+2 (33+1–36+4)	39+6 (39+1–40+2)	
Birth weight (grams)	2789 (2120–3229)	3500 (3255–3891)	
*Matching variables*			
Maternal age at sampling (years)	36 (33–39)	36 (33–40)	0.863
Nulliparous	12 (60.0%)	24 (60.0%)	1.000
IVF	1 (5.0%)	2 (5.0%)	1.000
Gestational age at sampling (weeks+days)	15+5 (15+0–16+1)	15+5 (15+2–16+0)	0.735
*Other variables*			
Maternal BMI at first prenatal visit	25.9 (21.3–28.1)	23.2 (21.8–26.7)	0.327
Smoking at first prenatal visit	2 (10.0%)	5 (12.5%)	1.000
Previous preterm delivery	3 (15.0%)	4 (10.0%)	0.676
Mode of delivery			
Vaginal delivery	12 (60.0%)	27 (67.5%)	0.566
Vacuum extraction	1 (5.0%)	4 (10.0%)	0.656
Cesarean section	7 (35.0%)	9 (22.5%)	0.302
Neonatal sex			1.000
Male	10 (50.0%)	20 (50.0%)	
Female	10 (50.0%)	20 (50.0%)	
Apgar score < 7 at 5 min	0 (0.0%)	1 (2.5%)	1.000

Continuous variables were analyzed using a Mann-Whitney *U* Test and are presented as the median (interquartile range; IQR). Categorical variables were analyzed using Pearson Chi-Square or Fisher’s Exact Test (when ≤5 individuals) and are shown as N (%).

NGAL concentrations (spontaneous PTD: median 398.0 ng/mL (IQR 165.7–502.0) vs term: median 325.5 ng/mL (IQR 228.3–499.8); *p* = 0.448) and PAI-1 concentrations (spontaneous PTD: median 41.4 ng/mL (IQR 26.4–54.8) vs term: median 40.1 pg/mL (IQR 22.4–59.4); *p* = 0.718) were not significantly associated with spontaneous PTD in the replication phase (Table 6). Neither were there any associations between the mid-trimester amniotic fluid concentrations of NGAL (r = 0.05; *p* = 0.722) and PAI-1 (r = -0.10; *p* = 0.443) and gestational duration.

**Table 6:** Distribution of protein concentrations among cases and controls in the replication phase.

Short protein/ gene name	Spontaneous preterm delivery (*n* = 20)	Term delivery (*n* = 40)	*p*
NGAL (ng/mL)	398.0 (165.7–502.0)	325.5 (228.3–499.8)	0.448
PAI-1 (ng/mL)	41.4 (26.4–54.8)	40.1 (22.4–59.4)	0.718

Protein concentrations are shown as median (interquartile range; IQR). *p* values are obtained with a linear mixed model on log transformed values and with matched set as random factor.

## Discussion

Studies of amniotic fluid have the potential to provide insights into basic biological mechanisms [12] of a wide range of pregnancy complications [12, 30] and to increase our understanding of key biological processes within the amniotic cavity. In a well-controlled study of mid-trimester amniotic fluid samples, we found a significant association between a lower quantity of NGAL and PAI-1 with spontaneous PTD and a shorter gestational duration in the first cohort, but not in the second, independent cohort. In the exploratory phase, 33 proteins met the final filtering criteria of which nine proteins (EC-SOD, IGFBP-5, IGFBP-7, LCN15, MFAP4, NGAL, PAI-1, SEMA A (V) and U-II) were associated with spontaneous PTD and gestational duration adjusting for gestational age at sampling. However, none of the proteins from the exploratory phase were significant after correction for multiple testing.

The onset of labor, both at term and at preterm, is initiated by inflammatory processes [31, 32] by a common pathway involving uterine contractility, cervical ripening, decidual and fetal membrane activation leading to rupture of the chorioamniotic membranes [33]. One fundamental difference is though that term labor results from a physiological activation of the components of the common pathway, while preterm labor arises from pathologic processes or stimulus that activate one or more of the components of the common pathway of parturition, causing an abnormal or disturbed regulation of the maternal inflammatory response. This may lead to an overproduction of cytokines, chemokines, prostaglandins and proteases [6] but could similarly cause a dysregulation with pre-term labor as the resultant pathophysiology.

NGAL, also known as Lipocalin 2 [34], is expressed by neutrophils and has the ability to bind to small extracellular molecules and substances (e.g. lipophilic molecules [35], retinol, prostaglandins, fatty acids, steroids, iron, and matrix metalloproteinases (MMPs)). Notably, NGAL expression is *higher* in the amniotic fluid and trophoblast cells in the late second and third trimester of women with PTL [27, 36, 37]. In our study, we analyzed samples in the early second trimester that predated the diagnosis of PTL by weeks to months. An association between *lower* levels of NGAL and spontaneous PTD in the mid-trimester is interesting, because sequestration of iron is a key function important in limiting bacterial growth [38, 39]. An impairment in NGAL might increase the bioavailability of iron in the lower genital tract and predispose to an ascending infection and spontaneous PTD.

A few of the other candidate proteins associated with spontaneous PTD and gestational duration in the exploratory phase have also been described in studies of spontaneous PTD or inflammation. After delivery, a higher concentration of both PAI-1 and LCN-15 has been found in preterm infants, PAI-1 has also been observed in higher levels in the lungs of preterm newborns with respiratory distress syndrome [40]. PAI-1 is a serpine protease inhibitor involved in the cascade that leads to MMP degradation; preventing excessive MMP degradation is important for limiting tissue injury during infections [41]. Although *higher* levels of PAI-1 are detected in preterm infants after birth, a *lower* level of PAI-I in the mid-trimester may predispose to spontaneous PTD by allowing excessive MMP-associated tissue injury of the chorioamniotic membranes and facilitate microbial invasion of the amniotic fluid. In addition, IGFBP-5 seems to play a key role in controlling cell senescence and cell inflammation [42] and an increased activity of IGFBP-7 has been associated with cellular senescence and tissue aging [43]. Tissue aging is caused by yet uncharacterized biomolecular pathways of oxidative damage in the placental and fetal membranes and is related to spontaneous PTD and PPROM [44]. Overall, there is biological rationale for some of these candidate proteins to be linked to spontaneous PTD or inflammation, but their roles in mid-trimester amniotic fluid need further investigation.

Mathematical and statistical criteria used to filter and select the potential candidates were designed to maximize the chances to identify proteins with the greatest separation between women destined to have a spontaneous PTD and women who delivered at term, but whether this gestational time frame is the optimal timing for detection of early inflammatory processes that leads to the subsequent development of spontaneous PTD remains unknown.

The major strength of this study is the strong methodology, including the thorough selection criteria for cases and controls, the standardized protocols for sample processing and the three stages experiment with an exploratory phase followed by a validation phase and a replication phase using a second cohort. The cohorts used in this study are unique due to their relatively large number of healthy, asymptomatic women with subsequent spontaneous PTD, large cohorts of healthy women with a delivery at term, and a very low lost to follow-up rate. The structured and rigorous process of validating the kits for the use of amniotic fluid and carefully select the ones with the most potential should also be considered as strength.

Limitations of this study are that the enrolled women may not reflect the general population as they are of a more advanced maternal age and have a higher risk of fetal chromosomal abnormalities than the overall population. Mid-trimester amniotic fluid samples can however only be collected in line with clear clinical indications, such as genetic testing, leaving few other opportunities to collect such samples. By excluding women with known or suspected fetal abnormalities from the study and women with confirmed anomalies from analysis, the bias that this introduce has partly been handled. Further, only women who understood Swedish were enrolled. The rate of spontaneous PTD rate differs between ethnicities, which can affect the generalization. The limitation of the control group to delivery between 38+0 and 41+6 gestational weeks was based on a case control design but is not optimal from the continuous perspective where gestational duration is studied. Finally, ELISA kits were considered unsuitable for the use of amniotic fluid for three potentially interesting candidates (SEMA A (V), IGFBP-5 and IGFBP-7) from the proteomics exploratory phase.

Detection of mid-trimester amniotic fluid proteins associated with birth timing would open up new possibilities for diagnostic assay development, which would mark a significant advance in obstetrics. Several of the nine proteins from the exploratory phase have previously been described as being associated with spontaneous PTD etiology, but, to our knowledge, none of these proteins have previously been evaluated in mid-trimester amniotic fluid. This strengthened our decision to validate and replicate the findings even though they were not significant after correction for multiple testing. The novel findings of NGAL and PAI-1 as potential biomarkers for the subsequent development of spontaneous PTD and determination of gestational duration in the validation phase could not be replicated. It is therefore important to continue studies of asymptomatic women for the early identification of mid-trimester amniotic fluid proteomic markers of spontaneous PTD and gestational duration.

## Conclusions

Decreased concentrations of NGAL and PAI-1 were significantly associated with both subsequent spontaneous PTD and gestational duration but the findings could not be replicated in a second independent cohort. Further research is needed to determine the value of mid-trimester amniotic fluid proteins as potential predictive biomarkers of spontaneous PTD and gestational duration.

## Supporting information

S1 File Materials and methods. (PDF)

S1 Fig Proteomics workflow. After immunodepletion and protein digest, iTRAQ labeling and multiplexing of samples was performed followed by LC-MS/MS.
(TIF)

S1 Table Detailed overview of the 9 proteins subjected for validation. Data for this table is derived from the UniProt Consortium; ^a^ referred to as their short gene names (LCN15 and MFAP4).
(PDF)

S2 Table Commercial ELISA kits used to analyze protein levels in amniotic fluid. ^a^ samples that were under or above the detection limit in the given dilution, were re-analyzed in the dilution 1:10 or 1:100, respectively; ^b^ samples that were under the detection limit in the given dilution, were re-analyzed in the dilution 1:5 and 1:2,5. None of the samples were above the detection limit; ^c^ samples that were under the detection limit in the given dilution, were re-analyzed in the dilution 1:5. None of the samples were above the detection limit; ^d^ samples that were under or above the detection limit in the given dilution, were re-analyzed in the dilution 1:100 or 1:2000, respectively; ^e^ samples that were under or above the detection limit in the given dilution, were re-analyzed in the dilution 1:10 or 1:100, respectively.
(PDF)

S3 Table The complete list of dysregulated proteins from the exploratory phase. This table shows the complete list of the 33 protein groups that were dysregulated from the exploratory proteomics phase filtered by a quantitative difference (Δ) of intensity ratio between cases and controls of |Δ|≥0.2. Bold text indicates statistical significance at p<0.05 using a two-sided alternative hypothesis. Proteins that significantly correlated with both spontaneous PTD (p*) using Student’s t-test and gestational duration (p**), using linear regression adjusting for gestational age at sampling, were subjected for validation.
(PDF)

S4 Table Correlation between the final proteins’ concentrations and gestational age at sampling in the validation phase. This table describes the correlation between the final log transformed proteins’ concentration and gestational age at sampling using Pearson correlation. Bold text indicate statistical significance at p<0.05 using a two-sided alternative hypothesis.
(PDF)

S5 Table Maternal and neonatal characteristics in the respective cohorts. Continuous variables were analyzed using a Mann-Whitney *U* Test and are presented as the median (interquartile range; IQR). Categorical variables were analyzed using Pearson Chi-Square or Fisher’s Exact Test (when ≤5 individuals) and are shown as N (%).
(PDF)
