# Supplementary material for: Mid-trimester amniotic fluid proteome’s association with spontaneous preterm delivery and gestational duration
Source: PLoS One. 2020 May 7;15(5):e0232553. doi: 10.1371/journal.pone.0232553 (PMC7205297; doi:10.1371/journal.pone.0232553)
Supplement: S1 File — (PDF) [file pone.0232553.s001.pdf]

Supplementary Materials and Methods

Immunodepletion

Individual amniotic fluid samples were processed for the immunodepletion procedure using the Multiple Affinity Removal System (MARS). The samples were subjected to buffer exchange into the MARS Buffer A (Agilent, Palo Alto, CA) using Amicon (Ultra-Centrifugal Filters Ultracel K; 3 kDa, Merck Millipore Ltd. Tullagreen, Ireland) filter according to the manufacturer's instruction. Briefly, 400 µg of protein from each sample was mixed with 2 ml of MARS buffer A and filtered in a centrifuge for 1 hour at 4°C and 7500 *g*. When the retenate was concentrated below 200 µl, a Hamilton syringe was used to precise asses the volume and transfer the retenate into a new tube and fill up with the MARS buffer A to the final volume of 200 µl. All samples were thereafter stored in the -80°C freezer until immunodepletion.

The Alliance 2695 HPLC system (Waters, Milford, USA) was used to perform the immunodepletion of the 14 most abundant proteins (albumin, IgG, antitrypsin, IgA, transferrin, haptoglobin, fibrinogen, alpha-2-macroglobulin, alpha-1-acid glycoprotein, IgM, apolipoprotein AI, apolipoprotein AII, complement C3, and transthyretin) according to the manufacturer instructions. Briefly, 190 µl from each sample was injected on the MARS column (MARS Hu-14, 4.6x100mm, Agilent, Palo Alto, USA) at a flow rate 0.125 ml/min of MARS buffer A. Approximately 3 ml of the flow-through fraction was collected between 5 and 22 minutes. The high abundant proteins were eluted with 100% of MARS buffer B at a flow rate of 1 ml. The column was then equilibrated with MARS buffer A again. The column heater was set at 22 ± 2°C and the UV detector for 280 nm. The whole method was adjusted for 44 minutes.

The eluate containing low abundant amniotic fluid proteins was collected and frozen at -80°C immediately upon collection. All depleted samples were buffer-exchanged for water to remove the MARS A buffer prior digestion using the 3 kDa cutoff Amicon filters by 3 subsequent steps by filtering in centrifugation at 4°C and 7500 *g* for twice 20 minutes and 55 minutes in the last step. The filter was always filled up with water after every filtering step. The final volume of the retentate was filled up with water to 140 ul and frozen at -80°C for further analysis.

Digest of the amniotic fluid samples

Unless noted otherwise, all chemicals were purchased from Sigma Aldrich, St. Louis, USA. Twelve µg of total protein from each sample was digested. Each sample was brought to 0.1% Rapigest (Waters, Milford, USA), 250 mM triethylammonium bicarbonate (TEAB) (pH 8,5)) and 5 mM tris- (2-carboxyethyl) phosphine (TCEP) and incubated 1 hour at 60°C. Samples were allowed to cool and S-methyl methanethiosulfonate (MMTS) was added to a final concentration of 10 mM and incubated for 10 min at room temperature. LysC (Promega, Madison, USA) was added at a 1:50 enzyme: total protein (w/w) ratio and incubated for 4 hours at 37°C. Finally, trypsin (Promega, Madison, USA) was added at a 1:50 enzyme: total protein (w/w) ratio and incubated overnight at 37°C.

Isobaric labeling and multiplexing of the samples

For the isobaric labeling, the iTRAQ kit (AB Sciex, Foster City, USA) was used. All samples were evaporated to dryness and dissolved in 24 µl of 250 mM TEAB. From each sample containing 12 µg of protein, 3 µg was taken for the preparation of the global internal standard (GIS). 20 multiplexes were prepared. Each multiplex contained 9 µg of GIS at the 114 iTRAQ channel and 9 µg of each individual amniotic fluid sample at iTRAQ channels 115, 116 and 117.

For labeling, the iTRAQ labels were spun down and mixed with 70 µl of ethanol. The labeling scheme was as follow in each of the twenty multiplexes: the label 114 was used for labeling the GIS aliquot, labels 115, 116 and 117 were used to label individual amniotic fluid samples in each multiplex. Upon combining all GIS and samples within individual multiplexes, all 20 multiplexes were concentrated below 50 µl. Individual samples were acidified using trifluoroacetic acid (TFA) (Sigma Aldrich) and incubated for 1 hour at room temperature to fully hydrolyzed RapiGest and centrifuged to pellet the hydrophobic part of RapiGest. The supernatant was desalted using solid phase extraction columns (SPE). The SPE (Empore, C18, Supelco, Sigma Aldrich) column was equilibrated with methanol, washed with 5% ACN with 0.1% TFA solution and loaded with a sample. The samples were eluted with 500 µl of 80% ACN with 0.1% TFA solution. All samples were evaporated to dryness and frozen at -20°C for further analysis.

High pH fractionation

For lowering of the sample complexity, the high pH fractionation was performed on the analytical UltiMate3000 HPLC system (Thermo Fisher Scientific, Waltham, USA). From each multiplex, 25 µl was taken and diluted with 2% ACN (LS-MS grade, Fluka), 20 mM ammonium formate (28% ammoniac, Sigma Aldrich) till final concentration of peptides was approximately 0.3 µg/µl. Peptides were separated in linear gradient formed by 2% ACN, 20 mM ammonium formate (as mobile phase A) and 80% ACN with 20 mM ammonium formate (as a mobile phase B) in 40 min gradient from 3-50% of mobile phase B at flow rate 0.3 ml/min on the Xterra MS C18 column (3.5 um, 2.1x100mm; Waters). A total of 70 µl of peptides was injected on the column, 32 fractions between 6–30 minutes were manually collected into the 96-well plate. The fractions were mixed into 8 fractions (1-9-17 and 25 into 1 fraction, 2-10-18 and 26 into fraction 2, etc). All 8 fractions were evaporated to dryness and frozen at -20°C for further analysis.

LC-MS/MS analysis

Amniotic fluid peptide separation was performed by the UltiMate 3000 HPLC system (Thermo Fisher Scientific). From each fraction, 0.5 µg was loaded on PepMap100 ViperTrap (3 um, 100 A, Thermo Fisher Scientific) pre-column for desalting. The desalting was performed by the loading pump gradient by 2% ACN, 0.1% TFA (load mobile phase A) and 100% ACN (load mobile phase B). Peptides were eluted on an analytical column Acclaim PepMap RSLC (75 um, 50 cm, C18, 2 um, Thermo Fisher Scientific) for separation in linear gradient formed by 2% ACN, 0.1 % FA (mobile phase A) and 80% ACN, 0.1% FA (mobile phase B), from 6 to 44 % of mobile phase B in 60 minutes at the flow rate of 200 nl/min. The MS analysis was performed on Q Exactive Plus (Thermo Fisher Scientific) in Information Dependant Aquisition (IDA) mode. MS spectra were acquired across the mass range of 350–1600 m/z in high-resolution mode (> 70 000) using 100 ms accumulation time per spectrum. A maximum of 10 precursors per cycle was chosen for fragmentation from each MS spectrum with 60 ms minimum accumulation time for each precursor and dynamic exclusion time for 8s. Tandem mass spectra were recorded in high sensitivity mode (resolution >17 500) with normalized energy collision (NCE) adjusted for iTRAQ samples at 33 %. All samples were analyzed in three technical triplicates. The sample sequence was the following: the blank was analyzed in the 10-min gradient (35 min method: 6% B – 45% B – 2% B) followed by the standard (100 fmol/µl CytC) analyzed in 30 min gradient (60 min method: 2% B – 45% B – 6% B) followed by 4 fractions of the sample analyzed in 60 min gradient (85 min method: 6% B – 45% B). The first replicate of all fractions was analyzed, the second and third replicate followed.

Data analysis

The following parameters were used to identify and quantify individual proteins in the Max-Quant software version 1.5.2.8 using the reverse decoy mode and integrated false

discovery rate (FDR) analysis: fixed modifications: methylthio; decoy mode: revert; special amino acids: KR; Include contaminants: True; MS/MS tol. (FTMS): 20 ppm; Top MS/MS peaks per 100 Da. (FTMS): 12; MS/MS deisotoping (FTMS): True; PSM FDR: 0.05; Protein FDR: 0.05; Site FDR: 0.05; Use Normalized Ratios For Occupancy: True; Min. peptide Length: 5; Min. razor peptides: 1; Min. peptides: 1; Peptides used for protein quantification: Razor; Discard unmodified counterpart peptides: True.

Survey MS and MS/MS spectra were processed in MaxQuant software v. 1.5.2.8 with following parameters: Methylthio (C) as fixed modification; revert decoy mode; lysine (K) and arginine (R) as special AAs; contaminants were included; MS/MS tolerance was set to 20 ppm; 12 (10?) Top MS/MS peaks per 100 Da. PSM, Protein and Site false discovery rate threshold was set to 0.05. Protein quantification was based on at least one unique or razor peptide with minimal length of 5 amino acids.

The primary data set was further filtered using two conditions: 1) a minimum of two valid values in the three replicates and 2) the coefficient of variance (CV) among triplicates was less than 20%. At the first stage, 58% of all values were removed (42% of values in the data set had 0 valid values in the 3 replicates; 16% had 1 valid value; 11% had 2 valid values and 31 % had all three valid values). Only 2% of the primary data set were filtered at the second step (2% of data had CV >20%; 4% of data had CV 15-20%; 11% of the data had CV 10-15%; 25% of data had CV 5-10%). Both filtering criteria were met in at least 75% in both groups of the primary data set (case and control).
