# Supplementary figures and images for: Mid-trimester amniotic fluid proteome’s association with spontaneous preterm delivery and gestational duration
Source: PLoS One. 2020 May 7;15(5):e0232553. doi: 10.1371/journal.pone.0232553 (PMC7205297; doi:10.1371/journal.pone.0232553)

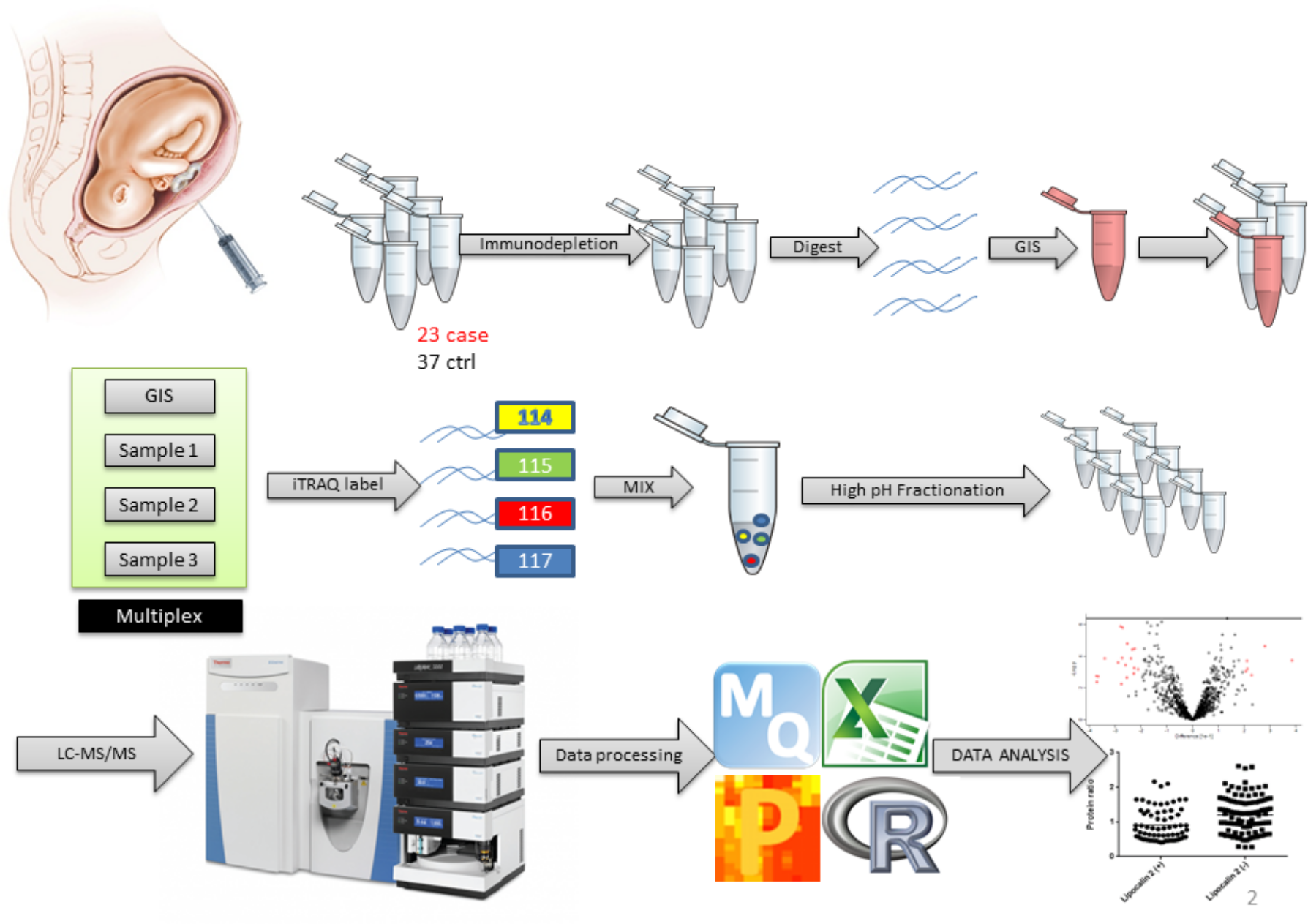

Supplement: S1 Fig — After immunodepletion and protein digest, iTRAQ labeling and multiplexing of samples was performed followed by LC-MS/MS. (TIF) [file pone.0232553.s002.tif]
