# Supplementary material for: Mid-trimester amniotic fluid proteome’s association with spontaneous preterm delivery and gestational duration
Source: PLoS One. 2020 May 7;15(5):e0232553. doi: 10.1371/journal.pone.0232553 (PMC7205297; doi:10.1371/journal.pone.0232553)
Supplement: S1 Table — Data for this table is derived from the UniProt Consortium; a referred to as their short gene names (LCN15 and MFAP4). (PDF) [file pone.0232553.s003.pdf]

Protein name	Primary accession number	Short protein name	Gene name	Short gene name
Extracellular superoxide dismutase [Cu-Zn]	P08294	EC-SOD	Superoxide dismutase 3	SOD3
Insulin-like growth factor-binding protein 5	P24593	IGFBP-5	Insulin like growth factor binding protein 5	IGFBP5
Insulin-like growth factor-binding protein 7	Q16270	IGFBP-7	Insulin like growth factor binding protein 7	IGFBP7
Lipocalin-15[a]	Q6UWW0	*Missing*	Lipocalin 15	LCN15
Microfibril-associated glycoprotein 4[a]	P55083	*Missing*	Microfibril associated protein 4	MFAP4
Neutrophil gelatinase-associated lipocalin	P80188	NGAL	Lipocalin 2	LCN2
Plasminogen activator inhibitor 1	P05121	PAI-1	Serpin family E member 1	SERPINE1
Semaphorin-3B	Q13214	SEMA A (V)	Semaphorin 3B	SEMA3B
Urotensin-2	O95399	U-II	Urotensin 2	UTS2
