# Supplementary material for: Mid-trimester amniotic fluid proteome’s association with spontaneous preterm delivery and gestational duration
Source: PLoS One. 2020 May 7;15(5):e0232553. doi: 10.1371/journal.pone.0232553 (PMC7205297; doi:10.1371/journal.pone.0232553)
Supplement: S2 Table — a samples that were under or above the detection limit in the given dilution, were re-analyzed in the dilution 1:10 or 1:100, respectively; b samples that were under the detection limit in the given dilution, were re-analyzed in the dilution 1:5 and 1:2,5. None of the samples were above the detection limit; c samples that were under the detection limit in the given dilution, were re-analyzed in the dilution 1:5. None of the samples were above the detection limit; d samples that were under or above the detection limit in the given dilution, were re-analyzed in the dilution 1:100 or 1:2000, respectively; e samples that were under or above the detection limit in the given dilution, were re-analyzed in the dilution 1:10 or 1:100, respectively. (PDF) [file pone.0232553.s004.pdf]

Short protein/gene name	Commercial Kit	Sample dilution	Standard curve range	Company
EC-SOD	Human SOD3 (Extracellular superoxide dismutase [Cu-Zn]) ELISA Kit	1:50[a]	0.625–40 ng/ml	Nordic Biosite, Täby, Sweden
LCN-15	LCN15 (Lipocalin-15) ELISA Kit (Human) (OKCA01335)	1:10[b]	18.75–1200 pg/mL	Aviva systems biology, San Diego, CA, USA
MFAP4	Human MFAP4 (Microfibril-associated glycoprotein 4) ELISA Kit	1:10[c]	0.313–20 ng/ml	Nordic Biosite, Täby, Sweden
NGAL	Human Lipocalin-2/NGAL Immunoassay	1:200[d]	0.003–0.040 ng/mL	R&D Systems, Inc., Minneapolis, MN, USA
PAI-1	Human Serpin E1/PAI-1, DuoSet R&D kit	1:10	0.31–20 ng/mL	R&D Systems, Inc., Minneapolis, MN, USA
U-II	Human UTS2/Urotensin II ELISA Kit	1:20[e]	15.6–1000 pg/ml	LSBio, LifeSpan, Seattle, WA Bioscience, Inc., USA
