# Supplementary material for: Mid-trimester amniotic fluid proteome’s association with spontaneous preterm delivery and gestational duration
Source: PLoS One. 2020 May 7;15(5):e0232553. doi: 10.1371/journal.pone.0232553 (PMC7205297; doi:10.1371/journal.pone.0232553)
Supplement: S3 Table — This table shows the complete list of the 33 protein groups that were dysregulated from the exploratory proteomics phase filtered by a quantitative difference (Δ) of intensity ratio between cases and controls of |Δ|≥0.2. Bold text indicates statistical significance at p<0.05 using a two-sided alternative hypothesis. Proteins that significantly correlated with both spontaneous PTD (p*) using Student’s t-test and gestational duration (p**), using linear regression adjusting for gestational age at sampling, were subjected for validation. (PDF) [file pone.0232553.s005.pdf]

Protein names of the leading protein(s)	Primary accession number	Δ	*p**	*p***
Increased protein expression in spontaneous preterm delivery vs. term delivery				
Apolipoprotein A-II; Proapolipoprotein A-II; Truncated apolipoprotein A-II	P02652	0.22	0.087	**0.039**
Chorionic somatomammotropin hormone 2; Chorionic somatomammotropin hormone 1	P0DML3; P0DML2; Q14406	0.21	0.075	**0.008**
Complement factor H-related protein 1	Q03591; Q9BXR6	0.22	0.138	0.056
Hemoglobin subunit gamma-2	P69892	0.27	0.298	0.863
Lipocalin-15	Q6UWW0	0.41	**0.039**	**0.009**
Microfibril-associated glycoprotein 4	P55083	0.23	**0.040**	**0.011**
Plasminogen; Plasmin heavy chain A;Activation peptide; Angiostatin; Plasmin heavy chain A, short form; Plasmin light chain B	P00747;Q15195; Q02325	0.21	0.078	**0.0048**
Unconventional myosin-IXa	B2RTY4	0.22	0.120	**0.022**
Decreased protein expression in spontaneous preterm delivery vs. term delivery				
Beta-galactoside alpha-2,6-sialyltransferase 1	P15907	-0.23	**0.024**	0.057
Catalase	P04040	-0.26	0.165	0.273
Cathelicidin antimicrobial peptide; Antibacterial protein FALL-39; Antibacterial protein LL-37	P49913	-0.25	0.052	0.162
Delta and Notch-like epidermal growth factor-related receptor	Q8NFT8	-0.26	0.123	0.208
Dermcidin; Survival-promoting peptide; DCD-1	P81605	-0.25	0.092	0.218
Extracellular superoxide dismutase [Cu-Zn]	P08294	-0.26	**0.004**	**0.003**
FERM and PDZ domain-containing protein 1	Q5SYB0	-0.23	**0.007**	0.061
Fibrinogen beta chain; Fibrinopeptide B; Fibrinogen beta chain	P02675	-0.21	0.167	0.387
Glutathione peroxidase 3	P22352	-0.24	0.053	**0.027**
Glycodelin	P09466; Q15431	-0.35	0.080	0.055
Insulin-like growth factor-binding protein 5	P24593	-0.22	**0.046**	**0.029**
Insulin-like growth factor-binding protein 7	Q16270	-0.32	**0.043**	**0.017**
Keratin, type II cytoskeletal 2 epidermal	P35908	-0.39	**0.037**	0.051
Neutrophil defensin 3; HP 3-56; Neutrophil defensin 2; Neutrophil defensin 1; HP 1-56; Neutrophil defensin 2	P59666; P59665	-0.29	**0.014**	0.088
Neutrophil gelatinase-associated lipocalin	P80188	-0.28	**0.044**	**0.030**
Plasminogen activator inhibitor 1	P05121	-0.29	**0.001**	**0.001**
Plastin-2	P13796	-0.21	**0.024**	0.157
Protein notum homolog	Q6P988	-0.21	0.228	0.104
Protein S100-A6	P06703	-0.23	**0.008**	0.068
Protein S100-A9	P06702	-0.20	**0.040**	0.172
Pyruvate kinase PKM	P14618; P30613	-0.36	0.068	0.072
Rho GDP-dissociation inhibitor 2	P52566	-0.25	**0.013**	0.084

Semaphorin-3B	Q13214	-0.23	**0.001**	**0.011**
Transketolase	P29401	-0.20	**0.034**	0.224
Urotensin-2	O95399	-0.22	**0.009**	**0.019**
