# Supplementary material for: Mid-trimester amniotic fluid proteome’s association with spontaneous preterm delivery and gestational duration
Source: PLoS One. 2020 May 7;15(5):e0232553. doi: 10.1371/journal.pone.0232553 (PMC7205297; doi:10.1371/journal.pone.0232553)
Supplement: S4 Table — This table describes the correlation between the final log transformed proteins’ concentration and gestational age at sampling using Pearson correlation. Bold text indicate statistical significance at p<0.05 using a two-sided alternative hypothesis. (PDF) [file pone.0232553.s006.pdf]

**Short protein/ gene name**	**r**	***p***
EC-SOD	-0.105	0.429
LCN15	0.113	0.393
MFAP4	-0.147	0.266
NGAL	-0.271	**0.038**
PAI-1	-0.106	0.426
U-II	0.131	0.324
