# Supplementary material for: Mid-trimester amniotic fluid proteome’s association with spontaneous preterm delivery and gestational duration
Source: PLoS One. 2020 May 7;15(5):e0232553. doi: 10.1371/journal.pone.0232553 (PMC7205297; doi:10.1371/journal.pone.0232553)
Supplement: S5 Table — Continuous variables were analyzed using a Mann-Whitney U Test and are presented as the median (interquartile range; IQR). Categorical variables were analyzed using Pearson Chi-Square or Fisher’s Exact Test (when ≤5 individuals) and are shown as N (%). (PDF) [file pone.0232553.s007.pdf]

Variable	Cohort (*n*=59)	Independent cohort (*n* =60)	*p*
Gestational duration (weeks+days)	39+1 (36+3 – 40+3)	39+1 (36+4 – 40+1)	0.669
Maternal age at sampling (years)	37 (35 – 38)	36 (33 – 39)	0.268
Nulliparous	18 (30.5%)	24 (40.0%)	0.279
IVF	5 (8.5%)	3 (5.0%)	0.491
Maternal BMI at first prenatal visit	24.4 (21.2 – 26.8)	24.1 (21.7 – 27.1)	0.793
Smoking at first prenatal visit	3 (5.1%)	7 (11.7%)	0.322
Previous preterm delivery	4 (6.8%)	7 (11.7%)	0.529
Gestational age at sampling (weeks+days)	15+5 (15+2 – 16+1)	15+5 (15+1 – 16+1)	0.786
Mode of delivery			
Vaginal delivery	47 (79.7%)	39 (65.0%)	0.074
Vacuum extraction	3 (5.1%)	5 (8.3%)	0.717
Cesarean section	9 (15.2%)	16 (26.7%)	0.127
Birth weight (grams)	3245 (2820 – 3600)	3305 (2964 – 3669)	0.546
Neonatal sex			0.644
Male	27 (45.8%)	30 (50.0%)	
Female	32 (54.2%)	30 (50.0%)	
Apgar score < 7 at 5 min	2 (3.4%)	1 (1.7%)	0.619
